# Supplemental N-3 Polyunsaturated Fatty Acids Limit A1-Specific Astrocyte Polarization via Attenuating Mitochondrial Dysfunction in Ischemic Stroke in Mice

**DOI:** 10.1155/2021/5524705

**Published:** 2021-06-09

**Authors:** Jun Cao, Lijun Dong, Jialiang Luo, Fanning Zeng, Zexuan Hong, Yunzhi Liu, YiBo Zhao, Zhengyuan Xia, Daming Zuo, Li Xu, Tao Tao

**Affiliations:** ^1^Department of Anesthesiology, Central People's Hospital of Zhanjiang, Zhanjiang, Guangdong 524045, China; ^2^Department of Anesthesiology, Affiliated Shenzhen Maternity and Child Healthcare Hospital, Southern Medical University, Shenzhen, Guangdong 518000, China; ^3^The Fifth Affiliated Hospital, Southern Medical University, Guangzhou, Guangdong 510900, China; ^4^Department of Medical Laboratory, School of Laboratory Medicine and Biotechnology, Southern Medical University, Guangzhou, Guangdong 510515, China; ^5^Department of Anesthesiology, Nanfang Hospital, Southern Medical University, Guangzhou 510515, China; ^6^State Key Laboratory of Pharmaceutical Biotechnology, The University of Hong Kong, Hong Kong, China; ^7^Department of Neurosurgery II, Central People's Hospital of Zhanjiang, Zhanjiang, Guangdong 524045, China

## Abstract

Ischemic stroke is one of the leading causes of death and disability for adults, which lacks effective treatments. Dietary intake of n-3 polyunsaturated fatty acids (n-3 PUFAs) exerts beneficial effects on ischemic stroke by attenuating neuron death and inflammation induced by microglial activation. However, the impact and mechanism of n-3 PUFAs on astrocyte function during stroke have not yet been well investigated. Our current study found that dietary n-3 PUFAs decreased the infarction volume and improved the neurofunction in the mice model of transient middle cerebral artery occlusion (tMCAO). Notably, n-3 PUFAs reduced the stroke-induced A1 astrocyte polarization both in vivo and in vitro. We have demonstrated that exogenous n-3 PUFAs attenuated mitochondrial oxidative stress and increased the mitophagy of astrocytes in the condition of hypoxia. Furthermore, we provided evidence that treatment with the mitochondrial-derived antioxidant, mito-TEMPO, abrogated the n-3 PUFA-mediated regulation of A1 astrocyte polarization upon hypoxia treatment. Together, this study highlighted that n-3 PUFAs prevent mitochondrial dysfunction, thereby limiting A1-specific astrocyte polarization and subsequently improving the neurological outcomes of mice with ischemic stroke.

## 1. Introduction

Ischemic stroke is caused by interruption of the blood supply to a part of the brain leading to the sudden loss of function, which now is one of the leading causes of death and disability worldwide [1]. As the most abundant and diverse glial cells in the brain, astrocytes are believed to play a crucial role in neuroinflammation and the pathogenesis of ischemic neuronal death. In the condition of an acute ischemic stroke, the proliferated reactive astrocytes in the peri-infarct areas are favourable for maintaining neuronal homeostasis. An increasing number of studies indicate that in the acute phase of ischemic stroke, astrocytes limit brain damage by activation and glial scar formation [2], modulate neuroinflammation by releasing cytokines [3], reconstruct the blood-brain barrier by reestablishment of the astrocytic water channels [4], and affect the neuron survival by metabolic substrates [5, 6] and signalling molecule transfer [7]. In addition to such functional diversity, the transcripts of reactive astrocytes are also different. According to the transcripts, the reactive astrocytes are further classified into A1 astrocytes and A2 astrocytes, which exhibit temporal and functional specificity in ischemic stroke [8]. After nerve injury, A1 astrocytes can release inflammatory cytokines and neurotoxins that induce cellular and neuronal apoptosis in the brain, while A2 astrocytes promote neuronal survival and tissue repair by secreting several trophic factors. Therefore, it is important to investigate the proliferation and function of these two reactive astrocyte subtypes in the acute phase of cerebral ischemia.

N-3 polyunsaturated fatty acids (n-3 PUFAs), mainly including docosahexaenoic acid (DHA) and eicosapentaenoic acid (EPA), are essential to human health [9]. The brain is highly enriched with the essential n-3 PUFAs, especially DHA, which plays a fundamental role in the normal development and function of the central nervus system [10]. DHA, a major form of n-3 PUFAs in the brain, cannot be generated in vivo, being supplied instead from a constant of food such as fish oil [11]. Diet supplementation of n-3 PUFAs is well documented to elevate brain levels of DHA. In the past decades, a series of epidemiological studies and clinical trials have suggested that increasing dietary intake or nutritional supplementation of n-3 PUFAs is closely associated with a reduced risk to or therapeutic effects in various neurological disorders [12, 13]. Diet supplementation of n-3 PUFAs exerts beneficial effects on ischemic stroke as well [14]. Poststroke n-3 PUFA therapeutic regimen protects against neuronal loss in the grey matter and promotes white matter integrity. N-3 PUFAs reduce the infarction volume by attenuating reactive oxygen species (ROS) activation in neurons [15]. Moreover, n-3 PUFAs exhibit neuroprotection by anti-inflammation relied on its modification of microglia/macrophage plasticity in cerebral ischemia injury [16]. DHA enhances macrophage phenotypic shift toward an anti-inflammatory phenotype to reduced central and peripheral inflammation after stroke. Begum et al. reported that DHA has protective effects in cultural astrocytes in vitro ischemia by suppressing calcium dysregulation and ER stress [17]. However, the effects and mechanism of n-3 PUFAs on astrocytes and its potential function in stroke have not yet been well reported.

Our present study evaluated the efficiency of n-3 PUFA supplement in the transient cerebral ischemia model and investigated the impact of n-3 PUFAs on astrocytes. Our results showed that n-3 PUFA feeding improved stroke outcomes during the acute phase of cerebral ischemia associated with astrocyte plasticity. Dietary n-3 PUFA supplement upregulated the transcripts of A1-specific astrocytes, which is related to mitochondrial damage-related oxidative stress.

## 2. Materials and Methods

### 2.1. Animals

Male C57/BL6 mice (22 ± 2 g, Specific Pathogen Free, 8 weeks old) were obtained from the central animal facility of Southern Medical University (Guangzhou, China). The animals were housed under standard conditions of light and dark cycles (12 h : 12 h, temperature 25°C) with free access to food and water. In addition, the cages were regularly cleaned. All the animal studies were carried out according to the approved protocols and guidelines of the Institutional Animal Ethical Care Committee of Southern Medical University Experimental Animal Centre.

### 2.2. Transient Middle Cerebral Artery Occlusion Model Establishment

Establishment of the transient middle cerebral artery occlusion (tMCAO) model has been described in our previous article [18]. Briefly, mice were anesthetized with continuous inhalation of sevoflurane (2%-5%); the inner and outer muscles of the sternocleidomastoid muscle were separated to expose and isolate the right common, external, and internal carotid arteries. Subsequently, the superior thyroid and occipital arteries were separated and cauterized using a preheated electrocautery to prevent bleeding. The model was established by inserting a monofilament (approximately 2 cm) from the external carotid artery to the middle cerebral artery, avoiding the pterygopalatine artery. After the monofilament was inserted for 1.5 hours of ischemia, the monofilament was gently pulled out to form reperfusion. The wound was disinfected with iodine and sutured. To test the beneficent effect of n-3 PUFAs in the mice model of tMCAO, the mice were fed with an n-3 PUFA-enriched diet (concentration of DHA reaching 39.6%) 7 days before tMCAO procedure, as previously described [19].

### 2.3. Cell Treatment

Primary cultural astrocytes were subjected to oxygen and glucose deprivation (OGD) followed by reoxygenation, to mimic the ischemic/reperfusion-like condition in vitro. To induce OGD, the primary astrocytes were incubated with glucose-free DMEM and placed within a hypoxic chamber which was continuously maintained with 95% N_2_ and 5% CO_2_ at 37°C to obtain 1% O_2_ for 5 h. OGD was terminated by replacing the medium to DMEM/F12 with 10% FBS for 12 hours. Cells incubated in DMEM/F12 with 10% FBS under a normoxic atmosphere were used as the normoxic control. The cells were pretreated with 20 *μ*M docosahexaenoic acid (DHA) (Sigma, USA) for 6 hours before OGD. Subsequently, the A1/A2-associated genes were assessed. In some cases, cells were pretreated with 2 *μ*M mitochondria-targeted antioxidant (Mito-TEMPO) (Merck, USA) for 24 hours.

### 2.4. Infarct Volume Analysis

TTC (2,3,5-Triphenyltetrazolium chloride) staining was used to reflect cerebral infarction as a percentage of brain volume. The mice were anesthetized and the integral brains were quickly obtained and cut into 2 mm tissue slices, then stained with 2% TTC for 5 minutes and soaked in 4% formaldehyde for 6 hours. The brain slices were arranged in order and photographed. The area of cerebral infarction was calculated using the Image J 1.52a (the red area indicated no infarction; the white area indicated infarction). The infarct area was calculated as the area of the nonischemic hemisphere minus the noninfarcted area of the ischemic hemisphere. Infarct volume = infarct area × thickness (2 mm). The percent of cerebral infarction was calculated using the following formula: The percentage of cerebral infarction = infarct volume/the volume of the nonischemic hemisphere × 100%.

### 2.5. Rotarod Test

Sensorimotor functions were accessed by rotarod test after stroke. All mice were trained for 2 days before the model establishment (each mouse was tested twice and the speed of rotation was 5 rpm for 10 minutes). During rotarod testing, the speed of rotation was accelerated from 5 to 15 rpm over 60 seconds with a testing period cut-off of 300 seconds for each trial and 2 total trials performed. The fall latency of each mouse was recorded and averaged. The experimenter was blinded to the treatments given to each mouse.

### 2.6. Neurological Scoring

The neurological evaluation was conducted based on the Garcia scale as illustrated in Supplementary Table 1. The Garcia scale was divided into 6 subjects, including spontaneous activity, symmetry in movement of 4 limbs, forepaw outstretching, climbing, body proprioception, and vibrissae touch. The mice demonstrated normal neurological functions were assigned as the highest score (18 scores), and the severe functional impaired rats were assigned as the lowest score (0 score).

### 2.7. Primary Astrocyte Culture

Astrocytes were harvested from both the cortices of C57/BL6 on postnatal day (P1 to P3). Briefly, the brain tissue was collected and digested with 0.25% trypsin and Dnase I (ROCHE, USA) in 37°C for 10 minutes. Then, cells were suspended in single cells and cultured in Dulbecco's Modified Eagle's medium (DMEM)/F12 with 10% fetal bovine serum which was heat inactivation in 56°C for 30 minutes beforehand, then incubated at 37°C and 5% CO_2_. The culture medium was then replaced twice a day.

### 2.8. Immunofluorescence Staining and Quantification

At 1 day after tMCAO, animals were euthanized and perfused with saline followed with phosphate-buffered saline (PBS) containing 4% paraformaldehyde (PFA, Sigma-Aldrich). Brains were removed and cut into 20 *μ*m frozen cryosections using a microtome. Brain sections were fixed for 10 minutes in 4% paraformaldehyde (Solarbio, China) at room temperature, then permeabilized and blocked with 0.5% TritonX-100 (Sigma-Aldrich) and 3% bovine serum albumin (BSA, Solarbio, China) for 1 h at room temperature. Next, the brain sections were incubated with primary antibodies against S100*β* (1 : 200 dilution, Proteintech, China) and C3 (complement 3) (1 : 250 dilution, Abcam, USA) at 4°C overnight. The brain slices were washed three times with PBS-Tween-20 (0.1% *v*/*v*) and were incubated for 1 hour at room temperature with fluorescently labelled secondary antibodies including FITC-conjugated goat anti-mouse IgG (1 : 100 dilution, Bioss, China), Cy3-conjugated goat anti-mouse IgG (1 : 100 dilution, Bioss, China), and Cy3-conjugated goat anti-rabbit IgG (1 : 100 dilution, Bioss, China). After washing, cells were counterstained with DAPI (Solarbio, China) and analyzed using laser-scanning confocal microscopy (LSM900, Japan). Immunopositive cell quantifications were performed with the software of Image J software 1.52a by an investigator who was blinded to the experimental design.

Primary cultural astrocytes were washed once with PBS, fixed for 30 minutes in 4% paraformaldehyde (Solarbio, China) at 37°C and permeabilized with 0.5% Triton X-100 (Sigma-Aldrich) for 10 minutes. After 5 min wash with PBS three times, the cells were blocked with 1% bovine serum albumin (BSA, Solarbio, China) for 1 hour at room temperature. Cells were incubated overnight at 4°C with primary antibodies against S100*β* (1 : 300 dilution, Proteintech, China) or C3 (1 : 300 dilution, Gentex, Switzerland). The cells were washed three times with PBS-Tween-20 (0.1% *v*/*v*) and were incubated for 1 hour at room temperature with fluorescently labelled secondary antibodies the same as brain section immunofluorescence mentioned above.

### 2.9. RNA Extraction and Quantitative Real-Time PCR (qRT-PCR)

Total RNA was isolated from primary astrocytes using TRIzol reagent (Thermo Fisher Scientific, United States) according to the manufacturer's instructions. Total RNA (1 mg) was used to synthesize cDNA using a PrimeScript RT reagent Kit with gDNA Eraser (TaKaRa, China). Expression of mRNA was determined by quantitative real-time PCR (qRT-PCR) using the TB Green Premix Ex Taq II (TaKaRa, China). QRT-PCR was performed on the ABI QuantStudio 6 flex (Applied Biosystems, United States). *β*-Actin expression was quantified as internal control for mRNA analysis. The primer sequences used in these analyses can be found in the Supplementary Table 2. The results of the analyses were calculated and expressed according to an equation (2^−ΔΔCt^) which provides the amount of the targets, normalized to an internal reference. Ct is a threshold cycle for target amplification. Each biological sample was tested in triplicate.

### 2.10. Western Blot

Astrocytes or brain tissues were digested in RIPA extraction buffer (Beyotime, China). Protein samples were separated by 8% SDS-PAGE and transferred onto PVDF (polyvinylidene difluoride) membranes (Millipore, United States) in tank transfer system (Bio-Rad, United States). Membranes were blocked with 5% nonfat milk in Tris-buffered saline containing 0.1% Tween-20 (TBST) for 1 hour, washed three times in TBST, and incubated overnight at 4°C with primary antibodies against C3 (1 : 1000 dilution, Abcam, USA) or *β*-actin (1 : 1000 dilution, Abcam, USA). After incubation with the HRP-conjugated goat anti-rabbit IgG secondary antibody (1 : 10000 dilution, Da-UN, China), immunoreactive bands were detected by enhanced chemiluminescence (Millipore, United States). The protein bands were quantitatively analysed using ImageJ software 1.52a.

### 2.11. JC-1 Analysis

Briefly, cells were washed with PBS and suspended in 1 ml fresh medium containing 1 *μ*M JC-1 for 20 minutes at 37°C in the dark. After washing with PBS twice, the fluorescence intensity was captured with an inverted fluorescence microscopy (Nikon, Japan). For red fluorescence, the fluorescence intensity was measured at Ex/Em: 525/590 nm. The green fluorescence intensity was measured at Ex/Em: 490/530 nm.

### 2.12. Mito-Tracker Staining

The cells were washed twice with PBS and labelled at 37°C for 30 minutes with 400 nM MitoTracker Green (Ex 490 nm/Em 516 nm). Cells were then washed with PBS and incubated with Hoechst 33342 (1 *μ*g/ml) for 10 minutes at room temperature. Fluorescence was detected on a Nikon A1R scanning laser confocal microscope (Nikon, Tokyo, Japan). Fluorescence was detected on a Nikon A1R scanning laser confocal microscope (Nikon Corporation, Tokyo, Japan). The images were analysed using an image analysis system (Image-Pro Plus, version 6.0) with the Mitochondrial Network Analysis (MiNA) toolset according to a previously article [20].

### 2.13. Mito-SOX Staining

For Mito-SOX staining, astrocytes were harvested and stained with 5 *μ*M dye for 10 minutes at 37°C. The stained cells were excited at 510 nm, and the emitted fluorescence was detected at 580 nm by flow cytometry or confocal microscope.

### 2.14. Statistical Analysis

Data are expressed as mean ± SD. Differences were evaluated by one-way analysis of variance (ANOVA; three or more groups). *p* < 0.05 was considered statistical significance. Statistical analyses were performed using SPSS 22.0 Statistics (IBM SPSS Statistics for Version 22.0, IBM Corp, North Castle, NY, USA).

## 3. Results

### 3.1. Dietary n-3 PUFA Supplement Improved Infarction and Functional Outcomes after tMCAO

**Figure 1:**
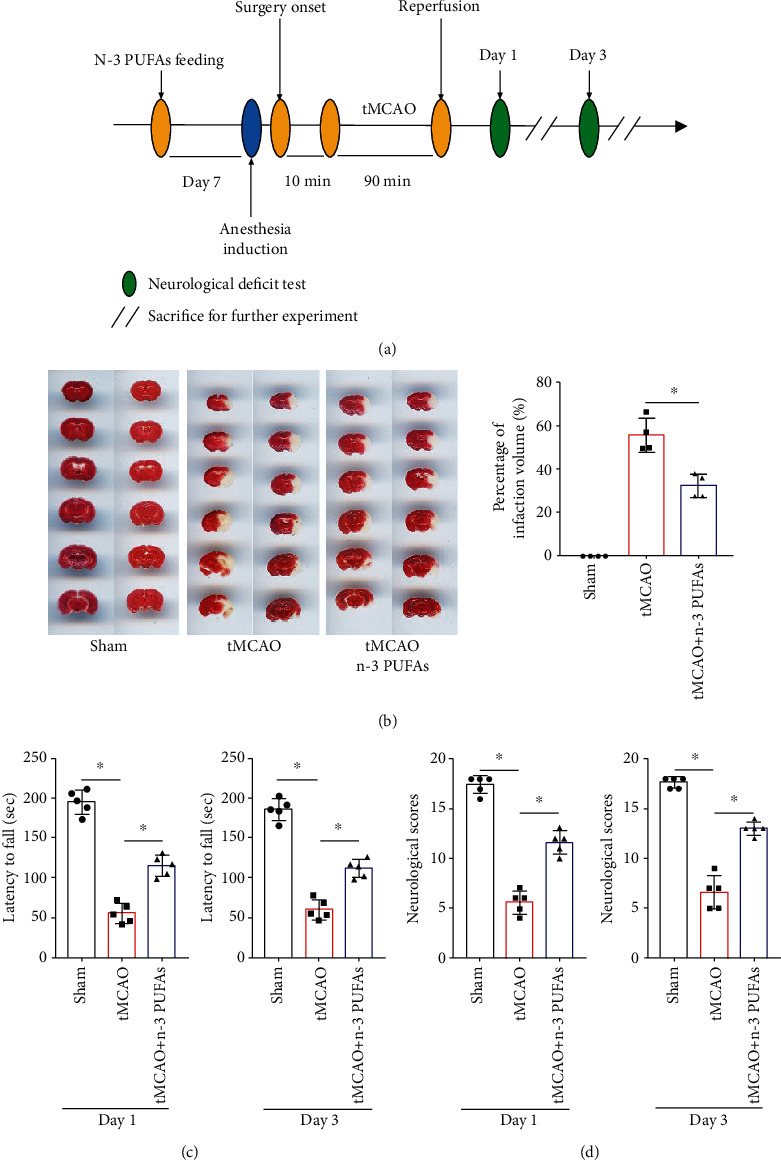
Effects of dietary n-3 PUFA supplementation on functional outcomes after tMCAO. (a) The schematic of experiments on male C57/BL6 mice in vivo. (b) 2,3,5-Triphenyltetrazolium chloride- (TTC-) stained brain slices from each group. (c) Sensorimotor functions were accessed by rotarod test after stroke. (d) Estimation of GARCIA neurological scores at day 1 and day 3 after tMCAO procedure. ^∗^*p* < 0.05.

**Figure 2:**
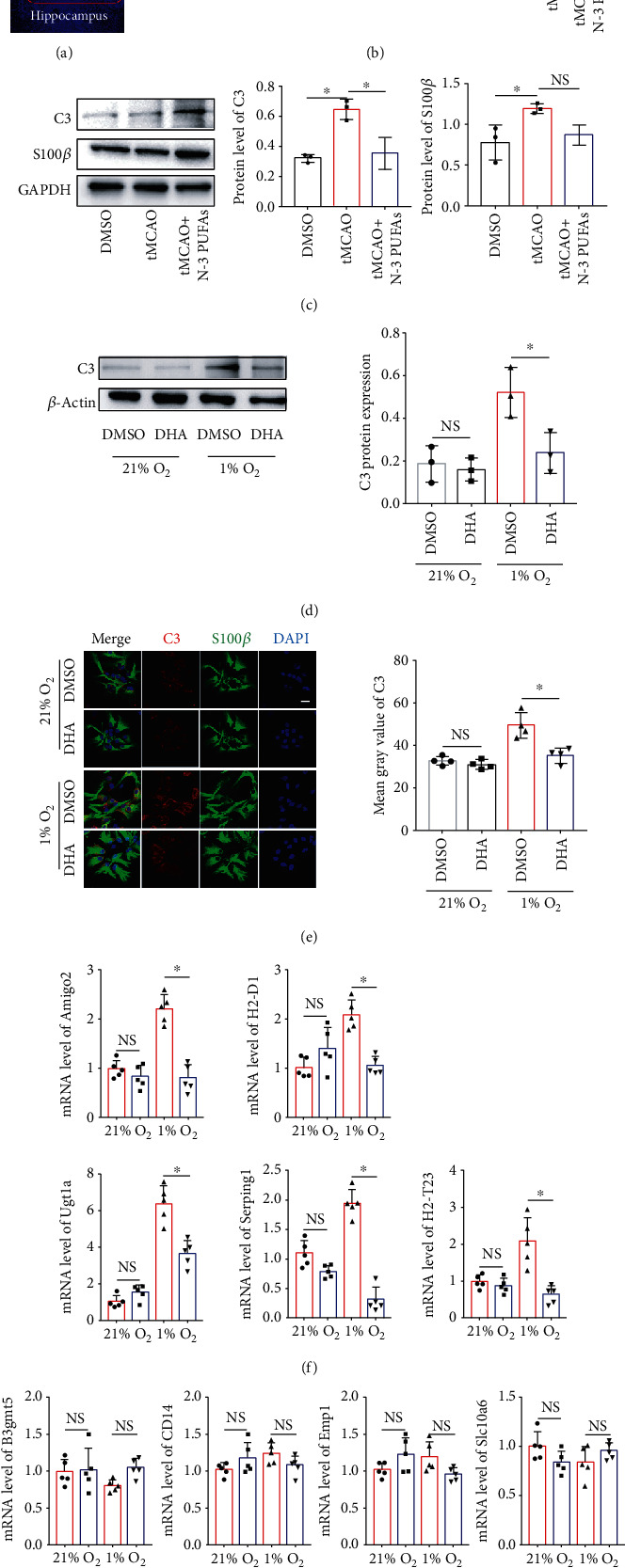
N-3 PUFAs influence the astrocyte polarization both in vivo and in vitro. (a) The brain regions of hippocampus were shown. (b) N-3 PUFA-fed mice and control mice underwent tMCAO procedure. The brain frozen cryosections were conducted with immunofluorescence staining for C3 and S100*β*. (c) The levels of C3 and S100*β* in brain tissue were evaluated using Western blot. (d–g) Primary astrocytes were cultured under 1% or 21% oxygen, respectively, followed by reoxygenation. The protein level of C3 in the astrocytes were determined by Western blotting (d) and immunostaining analysis (e) of C3 and S100*β* in primary cultural astrocytes. Additionally, the mRNA levels of A1 markers (f) and A2 markers (g) were evaluated by quantitative RT-PCR. ^∗^*p* < 0.05. One of the three independent experiments is shown. NS: not significant. Scale bar in 20x magnification = 100 *μ*m and scale bars in 80x magnification = 20 *μ*m (a). Scale bar in (e) is 20 *μ*m.

**Figure 3:**
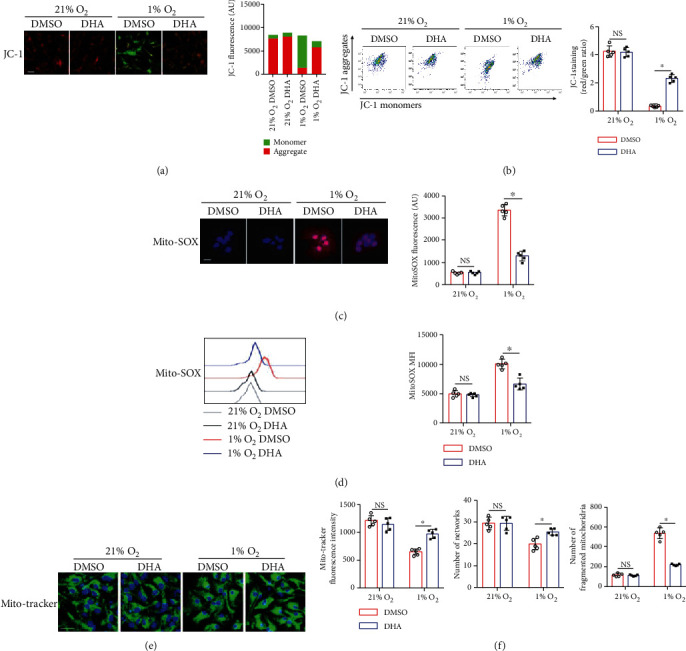
DHA protects against mitochondrial dysfunction in the astrocytes under hypoxia condition. Primary astrocytes were treated with DHA for indicated time periods under hypoxia condition. (a, b) The mitochondrial membrane potential in astrocytes was measured by the membrane-permeant JC-1 staining for confocal scanning (a) and flow cytometry analysis (b). (c, d) Mitochondrial ROS was measured by MitoSOX probes (c), and the MFI of MitoSOX (d) was calculated by Image J software. (e, f) MitoTracker was employed to mark mitochondria (e). Statistical analysis was performed on 200 cells in each group to obtain five data sets; quantification of mito-tracker fluorescence intensity, number of individuals, number of networks, and number of fragmented mitochondria were analyzed using the Image-Pro Plus with MiNA on mitochondrion-labelled images (f). Scale bar = 20 *μ*m. ^∗^*p* < 0.05. One of the three independent experiments is shown.

The experimental schedule was shown in Figure 1(a). In order to estimate whether dietary n-3 PUFAs reduce infarction and improve neurofunctional recovery after tMCAO, we used TTC staining, neurobehavioral test, and neurological scores to assess brain injury in mice. As shown in Figure 1(b), n-3 PUFA feeding significantly decreased the infarct volume in the mice model of tMCAO. The rotation duration of the sham group (194.8 ± 13.4 s and 185.2 ± 12.1 s) was longer than that of the tMCAO group (55.4 ± 10.1 s and 59.8 ± 11.2 s) on day 1 and day 3 after surgery (Figure 1(c)). Additionally, the neurological functions of n-3 PUFA-treated tMCAO mice improved significantly compared with the tMCAO mice (Figure 1(d)). Together, these results indicate a protective effect of dietary n-3 PUFAs in mice with cerebral ischemia/reperfusion injury.

### 3.2. N-3 PUFA Treatment Reduces A1-Specific Astrocytes Activation Both In Vivo and In Vitro

A great number of evidences pointed out that astrocytes play critical roles in the regulation of neurotransmission and neuron homeostasis and involved in the progression of acute CNS injury [21]. Recent studies reported that reactive astrocytes were distinguished into two types, A1 and A2 astrocytes [8]. We, therefore, raised a question whether n-3 PUFA affects the astrocytes polarization during cerebral ischemia/reperfusion injury. Immunofluorescence staining was employed to identify the level of complement 3 (C3), a particular A1 marker [8], on the S100*β*-positive astrocytes in hippocampus. The brain regions of hippocampus were displayed in Figure 2(a). As shown in Figures 2(b) and 2(c), the expression of C3 was increased significantly in the brain tissue from tMCAO mice compared to that from sham mice. Of note, n-3 PUFA treatment markedly suppressed the level of C3 in the mice model of tMCAO. To confirm the impact of n-3 PUFAs on astrocyte activation in vitro, primary cortex astrocytes were isolated and stimulation with or without DHA in the condition of low oxygen supply. The result displayed that DHA treatment limited the C3 expression induced by hypoxia in astrocytes (Figures 2(d) and 2(e)). Furthermore, our data showed that hypoxia treatment increased the mRNA expression of A1-specific markers (i.e., Amigo2, H2-D1, H2-T23, Serping1, and Ugt1a) in the primary astrocytes, and DHA treatment inhibited the upregulated levels of these A1 markers in the astrocytes under hypoxia (Figure 2(f)). By contrast, the mRNA expression of A2-specific markers (i.e., B3gmt5, CD14, Emp1, and Slc10a6) was not changed after DHA stimulation in the condition of low oxygen (Figure 2(g)). Together, these results suggest that n-3 PUFA administration reduces the A1 astrocyte polarization in mice with cerebral ischemia/reperfusion injury.

### 3.3. N-3 PUFAs Protect against Hypoxia-Induced Mitochondrial Dysfunction of Astrocytes

In astrocytes, mitochondria play an essential role in determining the cell fate. It has been reported that hypoxia induces impaired mitochondrial function and oxidative damage. We, therefore, investigated whether n-3 PUFAs influence the hypoxia-induced mitochondrial dysfunction in astrocytes. The result showed that DHA markedly reduced the hypoxia-induced mitochondrial dysfunction as indicated by increased mitochondrial membrane potential (Figures 3(a) and 3(b)) and reduced levels of mitochondrial oxidants (Figures 3(c) and 3(d)). Additionally, DHA treatment restored mitochondrial morphology under hypoxia conditions, as indicated by the mito-tracker fluorescence intensity, the number of networks, and the number of fragmented mitochondria (Figures 3(e) and 3(f)). These data indicate that n-3 PUFA treatment attenuates mitochondrial dysfunction and increase mitochondrial membrane potential in the astrocytes in the condition of hypoxia.

### 3.4. DHA Increases Mitophagy of Astrocytes under Hypoxia Condition

**Figure 4:**
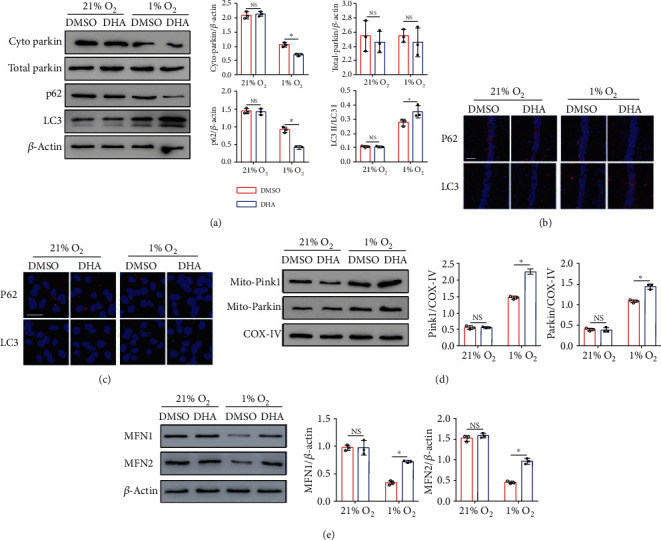
Effects of DHA on mitophagy in the astrocytes treated with hypoxia. Primary astrocytes were cultured at 21.0% or 1.0% oxygen in the presence or absence of DHA. (a) The expression of parkin, p62, and LC3 in the astrocytes was determined by Western blot analysis. The expressions of P62 and LC3 in brain tissue (b) and primary astrocytes (c) are shown by immunofluorescence staining. (d) The expression of mito-pink1 and mito-parkin in the mitochondrial fractions was determined by Western blot analysis. (e) The expression of mfn1 and mfn2 was detected by immunoblotting. Quantification was performed in images using the Image J software. ^∗^*p* < 0.05. Data from one representative experiment of three independent experiments are presented. Scale bar 100 *μ*m in (b), scale bar 20 *μ*m in (c).

Mitochondrial fusion and mitophagy are recognized as two critical processes underlying mitochondrial homeostasis. We investigated whether n-3 PUFAs affect mitochondrial fusion and mitophagy in astrocytes. DHA stimulation significantly decreased the expression of cytoplasmic parkin (cyto-parkin), but the total Parkin level was maintained, indicating that Parkin might translocate from the cytoplasm to the mitochondria (Figure 4(a)). Besides, the expressions of mitochondrial Parkin (mito-parkin) and pink1 (mito-pink1) were increased dramatically in the DHA treatment group compared with the control group in the condition of hypoxia (Figure 4(d)). Additionally, DHA treatment remarkably decreased p62 and increased LC3 under hypoxic condition both in brain tissue (Figure 4(b)) and primary astrocytes (Figures 4(a) and 4(c)). DHA significantly enhanced the expression level of mfn1 and mfn2 protein in the hypoxia-treated cells (Figure 4(e)). These results indicate that n-3 PUFAs promote mitophagy and mitochondrial fusion in astrocytes under hypoxia condition.

### 3.5. N-3 PUFAs Reduce the Astrocytes Polarization by Mitochondria-Targeted Antioxidation

**Figure 5:**
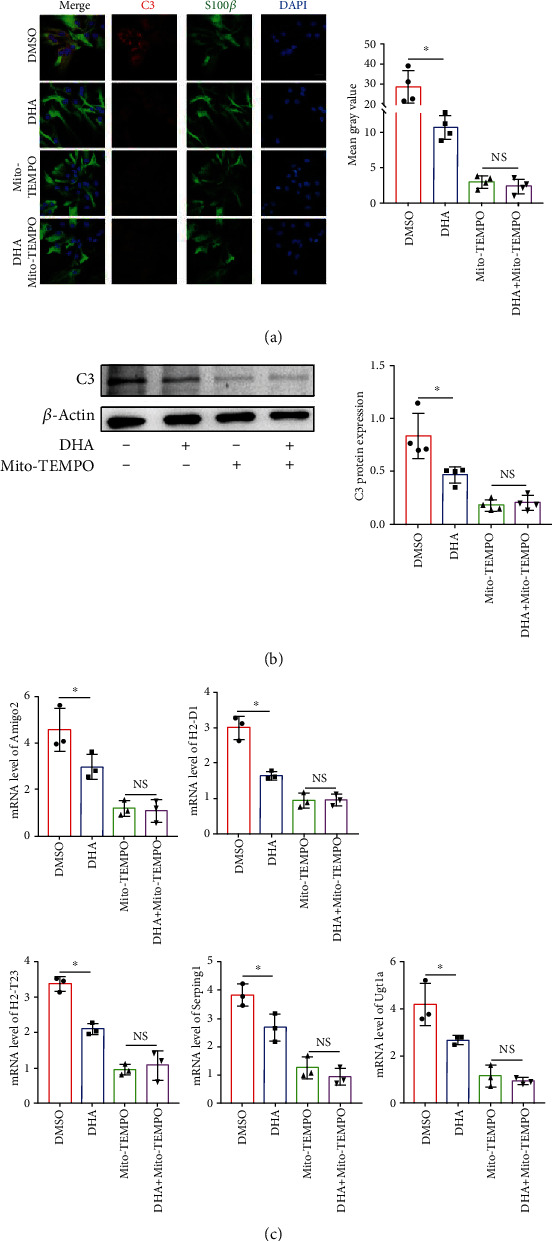
Mitochondria-targeted antioxidant abrogates the n-3 PUFA-mediated modulation of astrocyte polarization under hypoxia condition. Primary astrocytes were treated with DHA for indicated time periods under hypoxia condition. The mitochondria-targeted antioxidant and Mito-tempo were added 24 hours before the treatment. (a) The expression of C3 and S100*β* in primary cultural astrocytes was determined by immunofluorescence staining, and the integrated density was analyzed. (b) The protein level of C3 in astrocyte was evaluated by Western blotting. (c) The mRNA levels of the A1-specific markers were evaluated by quantitative RT-PCR. ^∗^*p* < 0.05; NS: not significant. One of the three independent experiments is shown. Scale bar in (a) is 20 *μ*m.

To explore whether n-3 PUFAs reduced A1 polarization through modulating the mitochondria dysfunction, we treated the astrocytes with a mitochondria-targeted antioxidant, Mito-TEMPO, along with DHA in the condition of hypoxia. Compared to DHA stimulation alone, the combination of Mito-TEMPO and DHA administration downregulated the mRNA level of A1 markers in the astrocytes with OGD/R treatment (Figure 5(a)). Moreover, DHA complexed with Mito-TEMPO significantly reduced the expression of C3 in astrocytes compared with DHA, as determined by Western blotting (Figure 5(b)) and immunofluorescence staining (Figure 5(c)). Upon Mito-TEMPO treatment, expressions of C3 and A1-specific transcript markers were comparable between DHA-treated and control cells (Figures 5(a)–5(c)). These results implied a possibility of DHA reducing A1-specific astrocytes polarization through the mitochondrial-derived oxidative stress.

## 4. Discussion

N-3 PUFAs play a critical role in the development and function of the CNS. In the current study, we determined that n-3 PUFA supplementation significantly decreased the infarction volume and improved the neurofunction after cerebral ischemia. Our data showed that n-3 PUFAs reduce stroke-induced A1 astrocyte polarization, probably via regulating the mitochondrial dysfunction, and exert anti-inflammatory and neuroprotective effects following ischemic stroke. Moreover, the n-3 PUFA-mediated modulation of mitophagy activity might be partially involved in the induction of A1 astrocytes.

Astrocytes undergo a transformation called “reactive astrocytosis” after cerebral ischemia stroke, whereby the transcription level of many genes upregulated [22, 23]. Functions of reactive astrocytes remain subjects of debate in ischemic brain injury, with previous studies showing that they can both hinder and support neurofunction recovery [22]. According to the different transcript expression profiles, astrocytes are classified into two major groups, A1-specific or A2-specific astrocytes [8]. Immediately after ischemic stroke, A1 astrocytes produce and secrete several proinflammatory mediators, such as IL-6, TNF-*α*, IL-1*α*, IL-1*β*, and IFN-*γ* [24, 25]. Additionally, A1 astrocytes can release neurotoxins that induce rapid death of neurons [3]. While in the later stage of stroke (after 72 hours), proliferation and glial scar formation of A2 astrocytes restrict the diffusion of neuroinflammation and produce neurotrophic factors [26]. The A2 astrocytes facilitate the generation of new blood vessels, protect neurons from excitotoxicity injury, and promote the formation of synapses [27]. Therefore, attenuation of the A1 astrocytes' polarization is supposed to reduce neuronal death and improve recovery from ischemic stroke. The current study shows that n-3 PUFA treatment markedly reverses the induction of A1 astrocytes caused by brain ischemia injury. Studies about astrocyte subtypes indicate that A1 astrocytes can be induced by IL-1*α*, TNF-*α*, and C1q secreted by LPS-stimuli microglia or other inflammatory signal pathways [8]. Joffre et al. reported that n-3 PUFAs have a suppressive effect on the production of proinflammatory cytokines in microglial cells, thereby resolving the brain inflammation and contributing to neuroprotective functions [28]. Therefore, it is possible that modulation of brain inflammation following ischemic stroke partially owing to the effect of n-3 PUFAs on microglia. However, DHA transported to brain tissues accumulates as a component of phosphatidylcholine or phosphatidylethanolamine in the cell membrane of astrocytes [29]. On the other hand, astrocytes have been shown to supply DHA to neurons and contribute to synapse formation, maturation, and maintenance [30]. It seems that the effects of n-3PUFAs on astrocytes are more direct. Considering that DHA has effects on both astrocyte and microglia, it is temporarily impossible to determine which cell plays a more critical role in ischemic brain injury based on the current experimental data. Thus, further investigations utilizing condition transgene mice aiming to astrocytes are warranted.

Astrocytes possess almost as many mitochondria as neurons to meet energy demands [31]. Of note, changes in astrocytic mitochondrial function are associated with the astrocyte activation in many neurodegenerative diseases. It has been reported that hypoxia and posthypoxia reoxygenation of primary astrocytes led to a drastic mitochondrial network change, followed by mitochondrial degradation and retraction of astrocytic extensions [32, 33]. Besides, mitophagy is essential for the quality control and homeostasis of mitochondria by eliminating dysfunctional mitochondria that produce reactive oxygen species (ROS) and result in cell death [34]. Mitophagy has fundamental connections with the mitochondria dynamic and is account for the various pathological stresses including stroke [12]. A previous study showed that supplementation with n-3 PUFAs exhibited mitochondrial phospholipid remodelling by increasing cardiolipin, a tetra-acyl phospholipid that is unique to mitochondrial and essential for optimal mitochondrial function [35]. DHA treatment after acute brain haemorrhage significantly attenuated mitochondrial disorder in neurons both in vivo and in vitro through preserving the mitochondrial morphology [36]. Similarly, our current research identified that DHA restored the mitochondrial junction disruption of astrocytes. Many studies reported that the damaged mitochondria accumulate under ischemic/reperfusion stress [37], suggesting that maintaining a pool of healthy mitochondria is crucial for protecting against tissue injury [33]. To ensure mitochondrial quality, mitophagy activated in the early stage of ischemic plays a significant role in the removal of damaged mitochondria [38], subsequently reducing the mitochondrial-associated neuron apoptosis or inflammation [39]. Our present data showed DHA pretreatment upregulated the protein levels of mitophagy-specific molecules in astrocytes at 12 hours after hypoxia, suggesting that the mitochondrial protection from ischemic/reperfusion stress by DHA is to some extent due to mitophagy enhancement. Another reactive mitochondrial quality control is reshaped by fusion and fission [34, 40]. The result demonstrated an upregulation of mfn1 and mfn2 (mitofusin-specific protein), which indicated that DHA promoted mitochondrial fusion in the hypoxia-treated astrocytes. It should be emphasized that mfn1 and mfn2 play a controversial part in the regulation of mitophagy [41]. Depletion of both mfn1 and mfn2 in murine cardiomyocytes caused the accumulation of defective mitochondria by inhibiting mitophagy [42]. In contrast, an active role of mfn2 in preventing mitophagy was also proposed, associating with the maintenance of ER-mitochondrial contacts [43]. Our current results were prone to support the opinion that DHA facilitated mitophagy through promoting mitofusin. Further, mitochondrial injury is known to contribute to oxidative stress in brain ischemic/reperfusion injury [44, 45]. DHA treatment also reduces oxidative stress by downregulating ROS and SOD in stroke [46]. Mitochondrial overproduction of ROS also initiates cell death and aberrant immune responses [47]. Among transcription factors, NF-*κ*B is induced in response to oxidative stress stimuli and participates in complex inflammatory loops regulating production and release of proinflammatory cytokines, such as tumor necrosis factor *α* (TNF-*α*) [48]. This proinflammatory cytokine is demonstrated to induce A1 astrocytes in previous studies [8, 49]. Thus, it is possible that n-3 PUFA participates in the polarization of A1-specific astrocytes through mitochondrial-derived oxidative stress and the inflammatory pathways.

## 5. Conclusions

In summary, we demonstrated that exogenous n-3 PUFA supplementation prevents mitochondrial-derived oxidative stress, resulting in limited A1-specific astrocyte polarization both in vivo and in vitro. N-3 PUFA-mediated blockage of A1 astrocyte polarization might be associated with the attenuated neuroinflammation in the brain ischemic/reperfusion stroke. These findings substantiate the concept that n-3 PUFAs have a potential clinical application to ameliorate ischemic/reperfusion brain injury.

## Data Availability

The raw data supporting the conclusions of this article will be made available by the authors, without undue reservation, to any qualified researcher.
