# Supplementary material for: Supplemental N-3 Polyunsaturated Fatty Acids Limit A1-Specific Astrocyte Polarization via Attenuating Mitochondrial Dysfunction in Ischemic Stroke in Mice
Source: Oxid Med Cell Longev. 2021 Jun 9;2021:5524705. doi: 10.1155/2021/5524705 (PMC8211499; doi:10.1155/2021/5524705)
Supplement: Supplementary Materials — The Supplementary Material for this article can be found online. See Supplementary Tables 1-2 in the Supplementary Material for comprehensive image analysis. [file 5524705.f1.pdf]

Supplementary Material

Supplementary Table 2 Garcia scales for scoring the neurological scores

Garcia scales	0 score	1 score	2 scores	3 scores
**Spontaneous activity**	Does not move at all	Barely moves in cage	Moved around reluctantly, reaches at least one side of cage	Moved around, explored cage
**Symmetry in movement of four limbs**	Forelimb on contralateral side does not move at all	Limbs on contralateral side exhibit minimal movement	Limbs on contralateral side extend less than those on ipsilateral side	All four limbs extend symmetrically
**Forepaw outstretching**	Left forelimb does not move at all	Left forelimb has limited movement	Left side outstretched less than left, forepaw walking impaired	Forelimbs outstretched, walk- ing symmetrically on forepaws
**Climbing**		Does not climb	Left side impaired, does not grip as tightly and releases before right	Climbs, grips tightly with both forepaws
**Body proprioception**		Does not react to stimulus on left side	Reacts slowly to stimulus on left side	Reacts by turning head, equally startled by stimulus on both sides
**Vibrissae touch**		Does not react to stimulus on left side	Reacts slowly to stimulus on left side	Reacts by turning head, equally startled by stimulus on both sides

Supplementary Table 1 Primers sequences used in current study

Genes	Sequence	Description
Amigo2-F	GAGGCGACCATAATGTCGTT	Genes of A1-specific astrocyte
Amigo2-R	GCATCCAACAGTCCGATTCT	
H2-D1-F	TCCGAGATTGTAAAGCGTGAAGA	
H2-D1-R	ACAGGGCAGTGCAGGGATAG	
H2-T23-F	GGACCGCGAATGACATAGC	
H2-T23-R	GCACCTCAGGGTGACTTCAT	
Serping1-F	ACAGCCCCCTCTGAATTCTT	
Serping1-R	GGATGCTCTCCAAGTTGCTC	
Ugt1a-F	CCTATGGGTCACTTGCCACT	
Ugt1a-R	AAAACCATGTTGGGCATGAT	
B3gnt5-F	CGTGGGGCAATGAGAACTAT	Genes of A2-specific astrocyte
B3gnt5-R	CCCAGCTGAACTGAAGAAGG	
Cd14-F	GGACTGATCTCAGCCCTCTG	
Cd14-R	GCTTCAGCCCAGTGAAAGAC	
Emp1-F	GAGACACTGGCCAGAAAAGC	
Emp1-R	TAAAAGGCAAGGGAATGCAC	
Slc10a6-F	GCTTCGGTGGTATGATGCTT	
Slc10a6-R	CCACAGGCTTTTCTGGTGAT	
